# Concurrent administration of a combined clostridial vaccine or ivermectin suppresses the antibody response to foot-and-mouth disease vaccine in sheep

**DOI:** 10.3389/fcimb.2026.1953270

**Published:** 2026-09-24

**Authors:** Shasha Wang, Limei Qin, Miaomiao Zhang, Xiangxiang Tan, Canyuan Li, Yuanhang Zhang, Ping Li, Han Gao, Shengfeng Chen, Feng Wen, Xingang Yu, Dajun Zhang, Keshan Zhang

**Affiliations:** Guangdong Provincial Key Laboratory of Animal Molecular Design and Precise Breeding, School of Animal Science and Technology, Foshan University, Foshan, China

**Keywords:** clostridial vaccine, foot-and-mouth disease vaccine, immune interference, ivermectin, sheep

## Abstract

Foot-and-mouth disease (FMD) is a highly contagious viral disease affecting cloven-hoofed animals and poses severe economic losses to the sheep industry. In intensive sheep farming, simultaneous administration of the FMD vaccine with other vaccines or anthelmintics is frequently practiced to reduce handling stress and labor costs. However, whether such combined administration can influence the immunological efficacy of FMD vaccine still lacks sufficient research evidence. In this study, 96 healthy sheep were randomly allocated into three groups (n=32 each). Group A received FMD vaccine alone (control); Group B received FMD vaccine concurrently with a quadrivalent clostridial vaccine; and Group C received FMD vaccine together with ivermectin. Serum samples were collected at 7, 14, 21 and 28 days post immunization, and liquid-phase blocking ELISA was applied to detect FMDV serotype O-specific antibody titers and the immune protection rate was determined based on whether the antibody titer exceeded 1:128. At day 28, the protection rates in Group A were 37.50% (12/32), while those in Groups B and C were only 0% (0/32) and 6.25% (2/32), respectively, with the ivermectin group showing significantly greater suppression than the clostridial group. In addition, both co-administration groups showed significantly lower titers at all time points. These findings demonstrate that concurrent administration of FMD vaccine with either quadrivalent clostridial vaccine or ivermectin severely impairs the humoral immune response to FMD in sheep, yielding inadequate protective levels. This study provides important field-relevant evidence for rational vaccine and anthelmintic application strategies in intensive sheep farming.

## 1 Introduction

Foot-and-mouth disease (FMD) caused by Foot-and-mouth disease virus (FMDV) is one of the most devastating animal diseases, affecting nearly all cloven-hoofed livestock including sheep, goats, cattle and pigs (Grubman and Baxt, 2004; Ma et al., 2022; Rahman et al., 2025). Infected sheep typically present clinical symptoms such as fever, lameness, foot cystic ulcerative lesions, weight loss and death, which severely restrict the trade of livestock products and causes significant economic losses to the sheep farming industry (Horsington et al., 2018; Stenfeldt et al., 2019; Yaser et al., 2023). Vaccination remains the most effective strategy for establishing herd immunity and preventing FMD outbreaks.

In intensive sheep farming, clostridiosis and parasitic infection are the two major disease threats alongside FMD. Ovine clostridial diseases are acute fatal infections induced by multiple pathogenic *Clostridium* species. Braxy, enterotoxemia, struck, and lamb dysentery represent major clinical disease syndromes of ovine clostridiosis, which are characterized by sudden onset and high mortality. Vaccination with quadrivalent clostridial inactivated vaccine is the core preventive measure against these diseases in China. Meanwhile, gastrointestinal and ectoparasites impair sheep growth and production performance. Ivermectin, a broad-spectrum macrolide anthelmintic, is widely applied for parasite control in sheep (Suárez et al., 2013; Martin et al., 2021). Notably, several *in vitro* studies have reported potential inhibitory effects of ivermectin on FMDV replication (Naeem et al., 2021; Arshad et al., 2023).

To reduce the stress and labor costs during the immune process on commercial sheep farms, the immunization against FMD, *clostridial* disease, and ivermectin deworming are usually carried out at the same time. Previous studies have indicated complex immunological interactions among vaccines, and between drugs and vaccines (Grubman and Baxt, 2004; Kabir et al., 2024; Wasfy et al., 2025). Nevertheless, it remains unclear whether simultaneous administration of quadrivalent clostridial vaccine or ivermectin interferes with FMD vaccine immune effect.

In this experiment, sheep were divided into three groups receiving FMD vaccine alone, FMD vaccine combined with quadrivalent clostridial vaccine, or FMD vaccine plus ivermectin. Serum samples were collected at 7, 14, 21, and 28 days post immunization, and the differences of FMDV serotype-O-specific antibodies were detected using liquid-phase blocking ELISA. We aimed to evaluate the impact of simultaneous clostridial vaccination or ivermectin treatment on FMD vaccine effect in sheep, providing experimental support for optimized vaccination and deworming schedules, and standardized disease prevention in intensive sheep farming.

## 2 Materials and methods

### 2.1 Experimental animals and management

A total of 96 healthy sheep, with a mean body weight of 18.0 ± 3.5 kg, half male and half female, aged 2 months, were selected from a commercial sheep farm after passing quarantine inspection. Prior to the experiment, all sheep were tested for FMDV serotype O antibodies to confirm that antibody titers were negative (< 1:8), and fecal examination was performed to confirm the absence of parasite ova, ensuring no history of FMDV infection and no recent use of immunosuppressive agents or anthelmintics. During the experimental period, all sheep were housed in separate pens under identical environmental conditions with ad libitum access to feed and water. The diet provided contained no antimicrobial drugs or anthelmintics.

### 2.2 Vaccines and drugs

The vaccines and drugs used in this study were obtained from domestic commercial manufacturers in China. The bivalent inactivated FMD vaccine (serotypes O and A) was injected intramuscularly at a single dose of 1 mL per sheep. The quadrivalent clostridial inactivated vaccine (against braxy, enterotoxemia, struck, and lamb dysentery) was administered intramuscularly at 2 mL per sheep. Ivermectin injection was given subcutaneously at the recommended dose of 0.2 mg/kg body weight according to the manufacturer’s instructions.

### 2.3 Experimental grouping and treatment

Considering the actual conditions of the sheep farm, the available number of experimental animals, and the capacity of the enclosures for breeding, the experimental sheep were randomly divided into three groups, with 32 sheep in each group (**Table 1**). Before conducting this experiment, no power calculation was performed. All individuals were marked for unified identification and regular feeding. Blood samples were collected at 7, 14, 21, and 28 days post-immunization for FMDV antibody titer detection.

**Table 1:** Grouping and treatment of experimental sheep.

Group	Sheep number	Blood collection time
FMDV vaccine	32	Blood was separately collected on days 7, 14, 21, and 28 after immunization.
FMDV vaccine + ovine *clostridial* vaccine	32	
FMDV vaccine + ivermectin	32	

### 2.4 Serum sample collection and processing

Blood samples (5 mL each) were collected from the jugular vein using vacuum blood collection tubes without anticoagulant at 7, 14, 21, and 28 days post-immunization. The collected blood was allowed to clot at room temperature for 1 h, then centrifuged at 3,000 rpm for 15 min. The serum was separated, aliquoted into 1.5 mL sterile centrifuge tubes, labeled, and stored at -20 °C until further analysis.

### 2.5 Detection of FMDV serotype O antibody titers

FMDV serotype O-specific antibody titers in serum samples were measured using a commercial liquid-phase blocking ELISA (LPB-ELISA) kit (Lanzhou Shouyan Biotechnology Co., Ltd., Lanzhou, China), strictly following the manufacturer’s instructions. The antibody titer of each serum sample was expressed as the reciprocal of the highest dilution yielding an optical density (OD) value above the 50% blocking threshold. According to the national standard for FMD vaccine efficacy evaluation, antibody titers ≥ 1:128 were considered indicative of clinical protective immunity, and animals meeting this criterion were defined as immune-qualified individuals.

### 2.6 Statistical analysis

All statistical analyses were performed using GraphPad Prism 5 software (GraphPad Software, Inc., San Diego, CA, USA). Antibody titer data were log_2_-transformed to approximate a normal distribution and were presented as mean ± standard deviation (Mean ± SD). Differences in antibody levels among groups at each time point were analyzed using one-way analysis of variance (One-way ANOVA). *P* < 0.05 was considered statistically significant.

## 3 Results

The FMDV serotype O specific antibody titers in serum samples collected at days 7, 14, 21, and 28 post-immunization were measured by LPB-ELISA. The antibody levels in Group A (immunized with FMD vaccine alone) reached the top at 7 day. On days 7, 14, 21, and 28 post-vaccination, 53.13% (17/32), 28.13% (9/32), 34.38% (11/32), and 37.50% (12/32) of sheep had antibody titers above the protective threshold, respectively (**Figures 1A–C**; **Table 2**).

**Figure 1:**
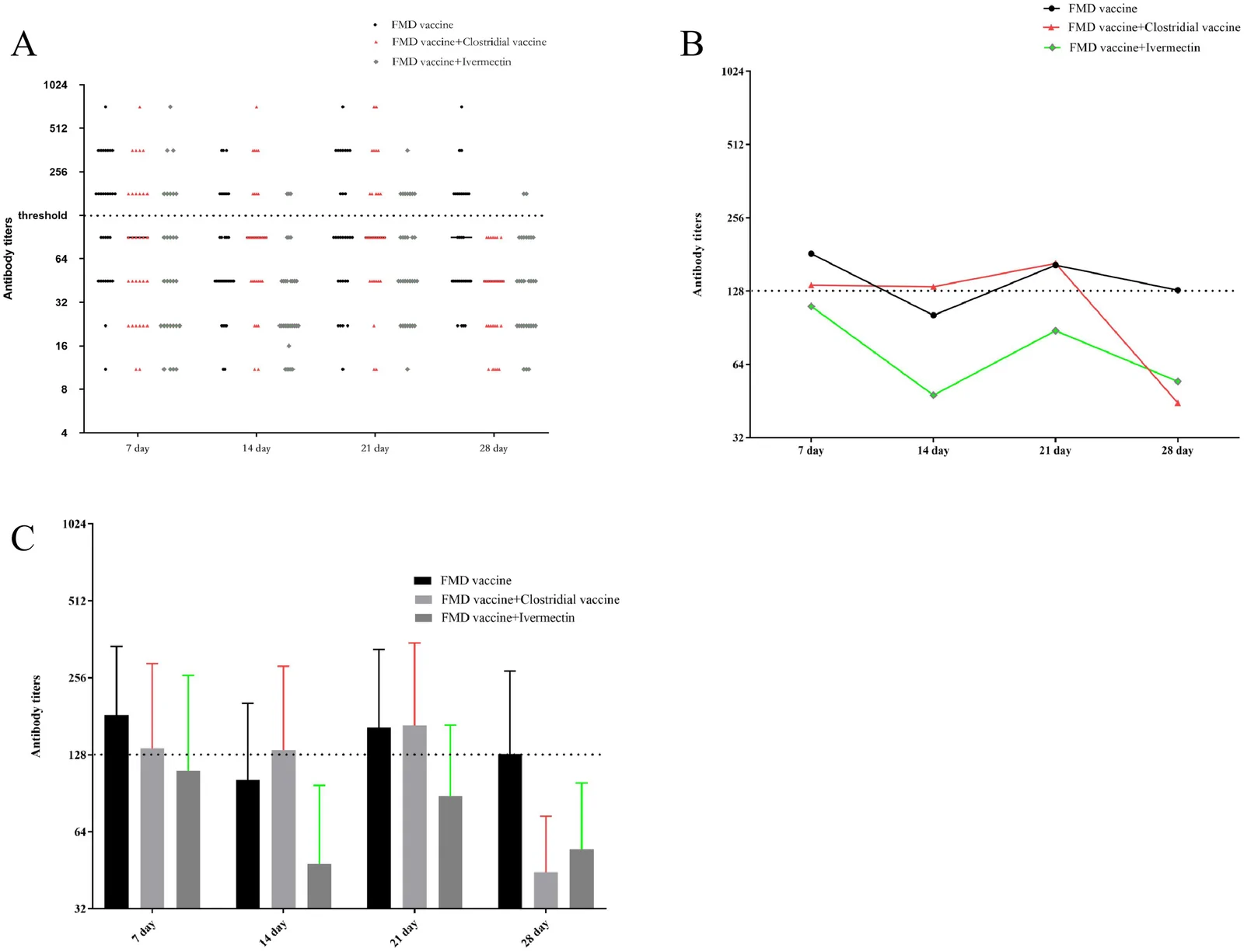
Dynamics of FMD-specific antibody titers in sheep following different vaccination treatments. **(A)** Scatter plot showing individual sheep O-type FMD antibody titers at the indicated time. Each dot represents antibody titer from one sheep. The dotted horizontal line indicates the seroprotection threshold titer. **(B)** Line graph illustrating mean antibody titers of three experimental groups at the indicated time. **(C)** Bar plot displaying mean antibody titers with standard deviation for each group at the indicated time. Black represents FMD vaccine alone group; red represents FMD vaccine combined with clostridial vaccine group; green represents FMD vaccine co-administered with ivermectin group.

**Table 2:** Statistics of positive rates in different groups.

Group	Parameter	Day 7	Day 14	Day 21	Day 28
A (FMD vaccine alone)	Protection rate (%)	53.13 (17/32)	28.13 (9/32)	34.38 (11/32)	37.50 (12/32)
B (FMD + *clostridial* vaccine)	Protection rate (%)	34.38 (11/32)	28.13 (9/32)	34.38 (11/32)	0 (0/32)
C (FMD + ivermectin)	Protection rate (%)	25.00 (8/32)	9.38 (3/32)	25.00 (8/32)	6.25 (2/32)

The antibody levels in Group B (immunized with FMD and quadrivalent clostridial inactivated vaccine at the same time) had a slight downward trend compared with Group A. And on day 7, 14, 21, and 28 after vaccination, 34.38% (11/32), 28.13% (9/32), 34.38% (11/32) and 0% (0/32) of sheep respectively (**Figures 1A–C**; **Table 2**).

The antibody levels in Group C (immunized with FMDV and Ivermectin at the same time) had a significant downward trend compared with Group A. And on day 7, 14, 21, and 28 after vaccination, 25.00% (8/32), 9.38% (3/32), 25.00% (8/32) and 6.25% (2/32) of sheep respectively (**Figures 1A–C**; **Table 2**). Altogether, as shown by the experimental data on the antibody levels and protection rates, concurrent administration of FMD vaccine with either a clostridial vaccine or ivermectin severely impairs the humoral immune response to FMD in sheep.

## 4 Discussion

FMD is an acute and highly contagious disease that is widely prevalent in regions such as the Middle East, Asia, and Africa (Grubman and Baxt, 2004; Kabir et al., 2024; Wasfy et al., 2025). Vaccination is recognized as the most economical and effective strategy for controlling FMD. In intensive sheep farming in China, farmers often administer the FMD vaccine concurrently with either the clostridial vaccine or ivermectin to reduce handling stress and labor costs. However, whether this concurrent practice affects the specific antibody response to the FMD vaccine remains insufficiently investigated.

In the present study, 96 sheep were divided into three groups to dynamically monitor serum FMDV serotype O antibody titers within 28 days post vaccination. Our results demonstrated that co-administration of quadrivalent clostridial vaccine or ivermectin significantly suppressed FMD-specific humoral immune responses at all sampling time-points. Both combined treatment groups exhibited lower antibody titers and reduced immune protection rates compared with sheep receiving FMD vaccine alone, and ivermectin produced stronger suppressive effects than clostridial vaccination, which is highly consistent with the research conclusions that multiple vaccines in livestock are subject to immune competition interference and inhibit the immunogenicity of a single vaccine (Sareyyüpoğlu et al., 2022; Göktuna and Çokçalışkan, 2025; Kim et al., 2026). These findings highlight that simultaneous implementation of multiple prophylactic interventions may impair vaccine-induced herd immunity in practical sheep farming.

The ovine quadrivalent clostridial vaccine is a multivalent inactivated vaccine that stimulates the production of specific antibodies against multiple toxin types of *Clostridium*. During simultaneous immunization, multiple antigens will compete for immune cells, cytokines, and immune response pathways in the body, thereby reducing the intensity of the immune response. In contrast, the FMD vaccine is mostly inactivated, with weak antigen stimulated ability and slower initiation of immune responses. In the case of multiple antigens competing for immune resources, the activation, proliferation, and differentiation processes of specific B cells are significantly inhibited, and the antibody secretion volume is greatly reduced, resulting in a decrease in antibody titer (Lee et al., 2020). At the same time, the non-specific immune responses induced by multi-component vaccines in the host can disrupt the immune microenvironment of the body, down-regulate the expression of immune-related cytokines, and further inhibit the efficiency of specific immune responses to FMD. This is one of the reasons why the immune effect of a single vaccine fails to reach the standard when multiple vaccines are administered simultaneously to livestock and poultry (Arzt et al., 2021; Kim et al., 2025).

This study has confirmed that the concurrent administration of ivermectin and the inactivated FMD vaccine can significantly suppress the specific humoral immune response of sheep against the FMDV, thereby reducing the vaccine protection efficacy. In addition to its well known anthelmintic and insecticidal activity, ivermectin has also been proven to exert immunomodulatory effects on both innate and adaptive immune responses by influencing the maturation of dendritic cells, the activation of T lymphocytes, and the balance of the cytokine network (Blakley and Rousseaux, 1991). Multiple studies have shown that ivermectin can inhibit lymphocyte proliferation, reduce macrophage phagocytic and processing functions, and selectively suppress the Th1-type cytokine production, thereby weakening the host ability to effectively recognize and respond to vaccine antigens (Stankiewicz et al., 1995). More importantly, during the critical initiation phase of the immune response after vaccination, ivermectin can interfere with the uptake of antigens by dendritic cells and the presentation by MHC-II molecules, subsequently inhibiting the function of FMDV-specific CD4^+^ T cells, the differentiation of plasma cells, and the secretion of IgG-type antibodies, as well as hindering the formation of memory B cells, ultimately resulting in dual impairment of the intensity and persistence of antigen-specific humoral immune responses (Dzhivhuho et al., 2018). The results of this study are consistent with those of several previous independent validations, confirming the conclusion that the combined use of anthelmintic drugs and vaccines has an immunosuppressive antagonistic effect, which can deepen the interpretation of the immune interaction mechanism under the combined intervention strategy for ruminants, providing theoretical basis and practical guidance for optimizing the timing scheme of anthelmintic and immunization in major disease prevention and control (Blakley and Rousseaux, 1991; Sajid et al., 2006; Rahmani et al., 2025).

Several limitations of the present study should be noted. Our experimental design lacked an unvaccinated blank control group and an ivermectin control group. Without these single control groups, we cannot fully separate the independent immune effects induced by clostridial vaccine or ivermectin alone, nor exclude the direct influence of ivermectin on serum readouts independent of FMD vaccination. Second, this investigation only evaluated humoral immunity by liquid-phase blocking ELISA to detect FMDV serotype O antibody titers. We did not assess cellular immune responses, cytokine profiles, or perform virus challenge experiments. Consequently, we can not fully characterize the comprehensive immune status of sheep or confirm actual *in vivo* protection against FMDV infection beyond serum antibody titers. Meanwhile, antibody responses induced by quadrivalent clostridial vaccine were not monitored, so it remains unknown whether FMD vaccination reciprocally interferes with the immunogenicity of clostridial antigens. Subsequent animal experiments will incorporate these additional control groups to further verify the immune interference phenomenon observed in this study.

## 5 Conclusion

In conclusion, our findings clearly demonstrate that concurrent administration of the clostridial vaccine or ivermectin with the FMD vaccine significantly suppresses the antibody response and reduces the protective immunity level in sheep. In the context of intensive sheep farming, we recommend avoiding the practice of scheduling multiple prophylactic interventions on the same day. Instead, vaccination and deworming programmes should be optimized by implementing staggered schedules to ensure the full efficacy of the FMD vaccine, thereby reinforcing the herd immunity barrier and minimizing the risk of disease outbreaks.

## Data Availability

The original contributions presented in the study are included in the article/supplementary material. Further inquiries can be directed to the corresponding authors.
